# Supplementary material for: Are carbon emissions trading and green financial instruments synergistic? -Comprehensive quantitative research based on content analysis
Source: PLoS One. 2024 Mar 7;19(3):e0298601. doi: 10.1371/journal.pone.0298601 (PMC10919601; doi:10.1371/journal.pone.0298601)
Supplement: S1 Data — (ZIP) [file pone.0298601.s001.zip › Data/地级市Z_F工作报告.zip!/╣≤╓▌/░▓╦│╩╨/└┤╫╘╣1⁄2╓┌║┼í╛┬φ┐╦╩2╛▌═°í┐_2005.docx]

各位代表:

现在，我代表市人民政府向大会作工作报告，请予审议，并请各位政协委员提出意见。

2004年政府工作回顾

2004年，市人民政府坚持以邓小平理论和“三个代表”重要思想为指导，全面贯彻党的十六大和十六届三中、四中全会，省委九届五次、六次全会和市委一届七次、八次、九次全会精神，牢固树立和落实科学发展观，在市委的领导下，在市人大、市政协的监督、支持和帮助下，紧紧依靠全市各族人民，着力实施农业稳市、旅游兴市、环境立市、工业强市战略和人才战略，认真执行国家宏观调控政策，努力克服固定资产投资减少、煤电运紧张对经济社会发展带来的困难和问题，紧紧扭住经济建设这个中心不动摇，团结奋进，锐意创新，经济保持了持续快速健康发展的良好势头，社会事业全面进步，人民生活不断改善。

一、主要预期目标圆满完成

全市生产总值87.79亿元，比上年增长10.1%，其中第一产业增长8.4%，第二产业增长10.6%，第三产业增长10.9%，三次产业构成为24.1：43.5：32.4。全社会固定资产投资24.60亿元，增长12.3%。财政总收入12.25亿元，增长21.9%，其中地方财政收入6.32亿元，增长18.8%。金融机构存款余额116.5亿元，增长21.4%；贷款余额122.6亿元，增长26.8%。社会消费品零售总额32.04亿元，增长13.0%。城镇居民人均可支配收入6752元，增长7.7%。农民人均纯收入1656元，增长5.1%。城镇登记失业率控制在4.4%以内。人口出生率和人口自然增长率分别控制在15.16‰和 9.09‰，分别比计划指标低1.34和0.91个千分点。

二、经济社会发展取得新成效

（一）坚持农业稳市，实现粮食增产、农业增效。加大对农业特别是粮食生产的投入，认真落实“一减三补”政策，全年粮食总产量75.63万吨，比上年增长2.4%。畜牧业发展步伐加快，总产值11.46亿元，增长16.2%，占农业总产值的34.3%。优质粮油、蔬菜、茶叶、烤烟、中药材等产业基地进一步扩大，“一乡一特，一村一品”成果得到巩固提高。油菜籽总产量7.12万吨，增长16.6%；蔬菜产量38.65万吨，增长7.7%；茶叶产量1017吨，增长70.1%；烤烟收购17.66万担，增长27%。新增5.12万农村贫困人口越过温饱线，完成易地扶贫搬迁1060户、4500人，解决农村1.2万户、6.6万人的饮水困难。新增有效灌溉面积3.01万亩，完成坡改梯4.1万亩，建沼气池7000口。加大对农村“土专家”的培训力度，5200人获得绿色培训证书。

（二）坚持旅游兴市，旅游业的龙头作用得到进一步发挥。成功举办首届中国贵州·安顺黄果树瀑布节。黄果树风景区游客首次突破百万人次大关，实现历史性跨越。成功举办了第二届贵州·安顺油菜花旅游节。格凸河申报国家级风景区工作进展顺利。启动了格凸河等风景区申报世界自然遗产工作。编制完成《安顺屯堡文化旅游区发展总体规划》、《安顺支线机场旅游线路规划》和《安顺旅游交通图》。黄果树景区、屯堡文化旅游基础设施建设进展顺利，黄果树石头寨和陡坡塘瀑布景点开发及二进龙宫等建设项目竣工。乡村旅游得到发展，有效增加了农民收入。安顺市游客服务中心投入使用。更加重视旅游宣传促销工作和外地主要客源市场的培育。全年接待国内外游客264.22万人次，实现旅游收入6.3亿元，分别比“非典”前的2002年增长89.1%、89.7%。旅游业的发展带动了餐饮、商贸、交通运输、通信、金融保险等相关服务业加快发展。

（三）坚持环境立市，发展环境不断优化。黄果树机场通航，清镇高速公路通车，镇胜高速公路全面开工建设，黄织铁路前期工作进展顺利。完成了100公里通乡油路和1000公里通村公路建设任务，通村公路覆盖率达到65.4%。王二河水库如期下闸蓄水，光照电站顺利截流，贯城河一期河道治理工程全面完成。黔中水利枢纽工程和马马崖、董箐梯级电站开发前期工作进展顺利。市、县区城镇规划和建设力度进一步加大，市级详规覆盖率达85%，完成各县《县城总体规划》修编及《近期建设规划》编制。城镇国有土地管理进一步加强。完成市区街、路、巷恢复历史称谓的命名工作。完成造林面积24.88万亩。石漠化治理规划编制工作全面展开。深入推进“建设诚信安顺、优化发展环境”活动，全面开展“群众评议机关”活动，发展环境进一步优化。扎实开展“卫生与秩序”创建活动，进一步巩固和扩大城镇环境综合整治成果，市区和县城环境卫生管理工作明显改进。大力开展食品药品安全、保护知识产权、农资打假等专项整治活动，市场经济秩序得到规范。认真开展环境管理体系认证工作，严格执行环境影响评价制度，环境保护机制初步建立。启动筹建市级政务服务中心。启动“十一五”规划编制工作。

（四）坚持工业强市，优势产业得到较大发展。加大力度调整工业经济结构。抓好现有煤矿的技改扩能和大中型煤矿建设，全年生产原煤752.48万吨，比上年增长26.7%，净增150万吨以上。完成发电量95.69亿千瓦时，增长12.9%；完成供电量31.1亿千瓦时，增长7.4%。地方优势产业得到较大发展，安顺电厂、百灵制药、牛来香、红蝶钡业、金星王啤酒、南风日化等企业竞争力不断增强。百灵制药等一批企业GMP改造项目通过验收，西秀制药厂等GMP改造启动实施。华泰一期技改顺利完成，贵州一家人油脂有限公司异地改扩建、明达水泥厂日产1000吨干法水泥等一批项目顺利开工建设。依托军工企业优势，高新技术产业有新的发展。全市规模以上工业完成增加值17.26亿元，增长13.6%。

（五）坚持实施人才战略，各项社会事业全面发展。高度重视人才工作，评选“市管专家”32名并发放专家津贴，对副高职称以上专业技术人员及研究生以上学历人员发放津贴。评出2002-2003年度市级科学技术进步奖23项，全年实施科技项目50项。完成了安顺市人才市场和劳动力市场建设。“安顺学院”申报前期工作进展顺利。安顺职业技术学院发展步伐加快。开展了培养“一村一名大学生”工作。镇宁、关岭“两基”工作，平坝“两基”复查工作通过省政府验收。全市“普九”人口覆盖率达到86.9%。完成“义教工程”等项目学校75所，新建和改造中小学校舍7.07万平方米。为全市农村16.4万名义务教育阶段贫困中小学生免费发放教科书。新建远程教育站点733个。进一步加大教育乱收费查处力度。加强公共卫生体系建设，疾病预防控制体系、卫生监督体系和医疗救治体系建设取得新进展。完成了乡镇卫生院管理体制上划工作。平坝县新型农村合作医疗试点工作进展顺利。农村计划生育利益导向机制初见成效。流动人口计划生育管理逐步规范。完成12个乡镇计生服务室改扩建和350个村计生服务室的建设任务。成功举办了“国庆文化周”等文化活动，花灯剧《屯堡人》被列为全省“五个一工程”重点剧目。改造和建设农村卫星广播电视收转站330座。完成关岭、紫云文化馆建设项目。民族地区经济发展，社会进步，召开了全市第一次民族团结进步交流暨表彰会。全市宗教稳定。完成市妇女儿童发展规划阶段性监测评估。体育、档案、史志等事业进一步发展。

（六）坚持深化试验区改革，全市改革开放步伐加快。深化贵州省多种经济成分共生繁荣试验区改革，把试验区改革扩大到全市。规范推进全市国有企业改制工作，国有企业改革基本完成年度预定目标。虹山轴承总公司政策性破产清算工作已终结、青松卷烟厂破产关闭工作进入准备阶段、市水泥厂政策性破产方案已上报，其他市、县区属国有企业改制工作抓紧进行。认真落实促进非公有制经济发展的各项政策措施，全市规模以上非公有制企业完成增加值6.66亿元，增长18.8%。按照《行政许可法》要求，深化行政审批制度改革，取消行政审批项目193项。完成了市县两级政府机构改革，事业单位人事制度改革试点工作顺利完成。农村税费改革、粮食购销体制改革进一步深化。市县两级全面推行国库集中收付制度，进一步加强部门预算，强化预算外资金管理，加强对财政资金的跟踪问效。金融部门不良资产率进一步降低，农村信用社改革工作进展顺利，城市信用社首次实现扭亏为盈，“政银合作”进一步加强。认真做好对口帮扶工作，加强对外交流与合作，全市招商引资实际到位资金9.48亿元，比上年增长61.1%，增加3.6亿元。

（七）坚持以人为本，认真解决关系人民群众切身利益的问题。全市新增就业12276人，其中下岗失业人员再就业4040 人（“4050”人员再就业576 人）。发放小额担保贷款409万元；减免再就业各种税费329.7万元；统筹就业培训9166人；劳务输出3.8万人，全面完成省下达任务。“两个确保”实现100%，“三条保障线”衔接良好，城市居民最低生活保障做到应保尽保并合理提高标准，工伤保险工作全面启动。全市共清理拖欠农民工工资403.6万元，减轻农民负担285.3万元。全市经济适用住房竣工4.86万平方米，第一期廉租房主体工程全部完工。认真抓好“两会一部”清欠兑付工作和水利水电工程移民工作，认真解决灾区、贫困地区和困难企业职工特别是困难企业军转干部生产生活问题，认真解决城镇房屋拆迁、企业改制等与群众利益密切相关的问题。做好矛盾纠纷排查调处工作，推进社会治安防控体系建设，加大社会治安综合治理力度，维护了社会稳定。加强自然灾害、重特大安全事故、重大食品安全事故等突发事件应急机制建设，维护了公共安全。安全生产实现“双降”目标。18件实事基本完成。

（八）坚持“三个文明”协调发展，社会主义民主政治和精神文明建设得到加强。自觉接受市人大及其常委会的法律监督和工作监督，认真向市人大报告工作；自觉接受市政协的民主监督，认真向市政协通报工作。进一步加大政务、村务、厂务公开力度，增强政策法规和工作的透明度。基层民主政治建设得到加强，社区建设工作进一步改进，集中解决了村干部报酬和社区居委会干部报酬，村（居）委会换届选举工作基本完成。深入贯彻《公民道德建设实施纲要》，大力加强和改进未成年人思想道德建设和大学生思想政治教育，实现全市公共文化设施和爱国主义教育基地向未成年人免费开放。深入推进文明单位、文明村寨、文明行业、文明窗口、文明社区及“五好文明家庭”创建活动，启动了文明机关、“四在农家”创建活动，全市城乡文明水平进一步提高。

三、工作措施进一步得到加强

一是坚持以科学发展观为指导，贯彻落实国家宏观调控政策，认真实施农业稳市、旅游兴市、环境立市、工业强市战略和人才战略，加大结构调整力度，促进经济增长方式转变，经济增长的内在活力不断增强、效益不断提高，国民经济发展的协调性进一步增强。二是围绕全年发展目标，紧紧依靠全市各族人民，创新工作思路，改进工作方式，狠抓工作落实，着力突破战略重点和关键环节，一批重大项目得到解决。三是统筹兼顾，注重加强农业等薄弱环节，切实加大对教育、卫生、计生、科技和社会保障等事业的投入，进一步加大对困难地区特别是一类重点扶贫乡镇的支持力度，努力实现经济社会之间、地区之间、城乡之间协调发展。四是把制约发展的难点作为深化改革的重点，进一步深化多种经济成分共生繁荣试验区改革，推进国有企业改革，推进行政管理体制、财政税收管理制度等改革，整合行政资源，从体制、机制和政策上增强发展的活力，着力营造良好的发展环境。五是全面开展“群众评议机关”活动，促进机关及机关工作人员作风转变和办事效能提高。六是增强市级统筹能力，充分发挥各县区独立发展的积极性，以城市为中心组织区域经济发展的格局逐步形成。

各位代表，2004年经济社会发展取得的成绩，是省委、省人民政府和市委正确领导的结果，是市人大和市政协监督、支持的结果，是各级各部门树立和落实科学发展观和正确政绩观、全面贯彻国家宏观调控政策的结果，是全市各族干部群众克服困难，艰苦奋斗，抢抓机遇，真抓实干的结果。在此，我代表市人民政府，向全市工人、农民、知识分子、各级干部和离退休老同志，向人大代表、政协委员，向各民主党派、工商联、人民团体、无党派人士，向驻安部队、武警官兵、政法公安干警和在安单位，向所有关心、支持安顺改革开放和现代化建设的各界人士致以崇高的敬意和衷心的感谢！

在肯定成绩的同时，我们清醒地看到，经济社会发展中还存在一些困难和问题。主要是：经济增长方式仍比较粗放，产业结构不尽合理；农业产业化程度低，农民增收的基础还不稳固，新阶段扶贫开发任务仍十分艰巨；工业化程度低，企业竞争力和盈利能力不强，国有企业改革和发展比较困难；就业和再就业空间小，社会保障水平不高；跨区固定资产投资增幅减缓，发展缺乏重大项目支撑；投资环境还需大力整顿，招商引资工作力度不够，招商引资方式需进一步改进；安全生产事故时有发生，维护社会稳定的任务依然艰巨，等等。

我们也清醒地看到，政府工作还存在不少缺点。政府职能转变滞后，行政审批手续繁锁，社会管理和公共服务不到位；一些部门职责不清，管理方式落后，办事质量不高，办事效率低下；一些部门之间协调不力，相互推诿，造成工作难以落实；一些关系群众切身利益的事情还没有得到解决；部分政府工作人员依法行政观念差，依法行政能力不强；一些地方、部门和单位存在着形式主义、官僚主义、弄虚作假的现象。对以上问题，我们将高度重视，并继续采取有力措施切实加以解决。

2005年政府工作主要任务

2005年是我市发展的关键一年，既要全面实现“十五”计划目标和撤地设市后首届市委、市人民政府任期目标，又要为“十一五”发展作好相关工作，任务艰巨而重大。胡锦涛总书记今年春节期间视察贵州发表重要讲话，要求贵州进一步抓住机遇、用好机遇，只要符合科学发展观的要求，有条件、有效益，就要加快发展，努力实现贵州经济社会发展历史性跨越。近年来全市广大干部群众共同努力保持的经济持续快速健康发展的良好势头，当前在开展保持共产党员先进性教育活动中广大党员干部群众焕发出的抓机遇、谋发展的强烈愿望和积极奋进、敢于争先的拼搏精神，以及基本形成的立体交通网络和人文资源、自然资源、人才技术、体制等方面的新优势，为我们深化市情认识，贯彻落实胡锦涛总书记的重要讲话精神，实现安顺经济社会发展历史性跨越奠定了良好的发展基础。我们要把智慧和力量凝聚到抓住机遇、用好机遇、加快发展上来，进一步解放思想、深化改革，紧紧抓住认准了的事情不放松，坚定信心，团结奋斗，善于创新，敢于进取，更好地团结和带领广大干部群众，推进安顺经济社会又快又好地发展，实现历史性跨越，把安顺建设成为繁荣、富裕、秀美、文明的新安顺。市委、市政府经过认真研究，制定了今年经济社会发展的总体要求和主要预期目标。

2005年政府工作的总体要求是：以邓小平理论和“三个代表”重要思想为指导，全面贯彻落实党的十六大和十六届三中、四中全会，省委九届六次全会，市委一届九次全会精神，用胡锦涛总书记今年春节期间视察贵州时的重要讲话精神统揽全年工作，以开展保持共产党员先进性教育活动为动力，更加坚定不移地贯彻发展是硬道理，更加坚定不移地贯彻发展这个执政兴国的第一要务，更加坚定不移地树立和落实科学发展观，认真落实中央加强和改善宏观调控的政策措施，进一步抢抓西部大开发和新阶段扶贫开发的机遇，深入实施农业稳市、旅游兴市、环境立市、工业强市战略和人才战略，继续推进社会主义物质文明、政治文明、精神文明建设与和谐社会建设全面发展，确保实现今年经济社会发展预期目标，全面完成和超额完成“十五”计划各项任务，努力推进安顺经济社会发展的历史性跨越。

2005年国民经济和社会发展的主要预期目标是：（1）全市生产总值增长10%以上，力争实现100亿元的历史性目标。（2）全社会固定资产投资增长12%以上，力争达到30亿元规模。（3）财政总收入和地方财政收入分别增长10%以上。（4）农民人均纯收入增加100元以上，实际增长5%以上；城镇居民人均可支配收入实际增长5%以上。（5）城镇登记失业率控制在4.6%以内。（6）人口出生率和人口自然增长率分别控制在15‰、9‰以内。

围绕全面完成和超额完成今年经济社会发展预期目标，重点要抓好以下几个方面：

一、进一步抢抓西部大开发机遇，千方百计保持固定资产投资较快增长

（一）大力推进西部大开发各项战略重点的实施。抓好机遇，用好机遇是我市实施西部大开发取得成效的关键，要以“西电东送”为重点，继续推进水、火、煤三位一体的能源建设。进一步做好安顺电厂三期工程项目前期工作，力争早日开工建设。继续配合做好光照电站建设和马马崖、董箐梯级电站建设的前期工作，加快三岔湾电站建设。继续抓好交通和水利基础设施建设。配合做好镇胜高速公路、黄织铁路建设。加快县际油路改造和通乡油路、通村公路建设，力争再上一批经济路、旅游路。继续配合做好国际一平方公里射电望远镜选址工作，尽最大的努力促成其落户普定尚家冲。配合做好黔中水利枢纽工程前期工作，进一步做好黄家湾水库工程前期工作，确保油菜河水库今年汛期满蓄，彻底做好王二河水库工程的扫尾工作。认真实施“滋黔工程”和“益民工程”。全面加强生态建设。继续抓好退耕还林、封山育林、天然林保护、珠江防护林体系等重点林业工程建设。进一步采取综合措施，解决退耕还林农户的长远生计问题和森林管护问题。充分发挥民兵预备役成建制参与生态建设的作用。建设一批高标准、高质量的沼气池示范村（乡）。尽快编制完成各县区喀斯特石漠化治理规划，修编市级治理规划，力争率先在全省完成规划编制，成为全省喀斯特石漠化治理先导性示范工程区。

采取有力措施，确保办实办好市委、市人民政府确定的与人民群众利益相关的“18件实事”。1、确保农民人均纯收入增加100元以上，实际增长5%以上。2、解决农村30000人的温饱问题。完成860户、4000人的易地扶贫搬迁。3、增加城镇就业岗位9900个，其中下岗失业人员再就业岗位3800个（“4050”人员再就业岗位300个）。组织实施农村劳动力就业转移培训16000人。4、为全市农村20万名义务教育阶段贫困中小学生免费发放教科书。5、进一步加强清理整顿农村合作基金会等“两会一部”的工作力度，采取多种方式解决好对储户遗留的资金兑付工作。6、解决14000户、65000人的饮水困难问题。建成农村沼气池13000口。7、完成90公里通乡油路和1200公里通村公路建设。8、完成 “义教工程”、“危改工程”、“农村寄宿制学校建设工程”54个。完成省下达的农村远程教育项目任务。9、完成272座农村卫星广播电视收转站的数字化改造，新建50户以上的自然村卫星广播电视收转站956座，实现70%的行政村通电话。10、新建或改扩建县、乡镇计生服务站9个，新建或改扩建村级计生服务室200个。改扩建10个乡镇卫生院和50个村卫生室。11、建成关岭宣传文化中心，在西秀、平坝、普定、镇宁、紫云5县区的一类扶贫重点乡镇建成农村精神文明活动中心，在全市6个一类扶贫重点乡镇符合条件的贫困村建成村级文化活动室。12、建设王若飞生平事迹陈列馆及停车场，建成双阳航空博览馆。13、完成塔山公园及地下停车场建设，完成市西路改造和西水路人行道改造。14、启动“引千入虹”工程、黄果树大街改造、虹山水库环湖路、南山路改造、市民健身中心建设项目，启动城区小街小巷治理改造工程。15、实施“平安安顺”工程。启动两城区公安派出所办公用房建设，实施城区规范化社区警务室建设工程，增加招聘城区100名巡防队员，为两城区派出所增配警务用车。16、承办好由省政府首次主办的中国贵州·安顺黄果树瀑布节，办好第三届贵州·安顺油菜花旅游节。17、组建完成市级政务服务中心。18、完成全市煤炭开采规划和9个煤炭勘查区的勘查工作，新增煤炭生产能力300万吨。

（二）全力做好项目工作。把进一步做好项目工作作为重大的战略性基础工程，组织专门力量切实加强项目调研、项目编制和项目储备工作。以项目促开发、促发展，按照“投产一批、续建一批、新开工一批、储备一批”的要求，抓好项目库建设，建立项目筛选、储备、发布及跟踪服务制度。认真研究国家和省的宏观经济政策和投资走向，尽快在农村基础设施建设、重大水利项目建设、教育基础设施、公共卫生设施、公检法司建设、生态建设和环境保护等领域准备一批大项目、好项目，争取国家和省进一步加大对我市的投入。高度重视“跑项目”，结合我市实际，着力在能源开发、旅游开发、生态农业、矿产资源开发、企业重组、新型工业、机械加工等方面组合包装一批对全市经济社会发展有重大影响的项目向外推出，并力求取得实效。

（三）进一步拓宽投融资渠道。在巩固与国家开发银行“政银合作”的基础上，进一步加强与金融机构的协调、沟通，争取金融机构扩大信贷投资规模。积极创新融资方式，探索采取产品支付、融资租赁、BOT以及债权融资等多种方式，扩大直接融资规模。抓住国家优先在西部地区进行产业投资基金试点的机遇，积极争取国家和省在我市旅游、能源、原材料等优势领域率先进行产业投资基金试点。认真贯彻执行国务院《关于投资体制改革的决定》，抓紧制定和出台我市的相关配套措施，加快推进核准制和备案制。进一步引导和支持民间资本参与各类开发和建设。对土地、矿产、林地、水等资源的出让收入以及各专项资金、基金进行全面清理，按照统筹使用，提高效益的原则，研究制定相应的管理办法和使用办法，提高政府投资调控能力。

（四）努力扩大招商引资规模。招商引资工作是安顺发展实现历史性跨越的关键，加强招商引资工作已成为我市加快发展的迫切需要。要规范招商引资工作制度，建立招商引资签约项目落实责任制，加强对重点项目、重点外商投资企业跟踪服务，提高招商引资项目签约率、开工率、投产率。鼓励和引导各类投资者参与我市国有企业资产重组、兴办初级产品特别是农产品深加工企业，并通过经营权转让和特许经营等多种方式，吸引投资者参与我市风景名胜区内项目以及市政公用设施等项目建设。按照“谁引进、谁受益”的原则，合理界定新引进企业的市、县区各级财政税收分成比例。通过委托招商、代理招商、专业化小分队招商、产业组团招商、定点定向招商、节庆招商、网上招商、以商招商、个人招商等方式，通过发挥民主党派、商会等联系广泛的优势，提高招商引资实效。有针对性地开展国内国际招商，特别是加强与我市经济互补性强的长三角、珠三角等地的“目的地”招商。抓好《安顺市招商引资鼓励办法》的落实，对符合国家产业政策及本市鼓励投资的新建工业项目、高新技术产业、观光酒店业等产业项目，切实落实好在用地、税收、行政收费等方面出台的优惠政策。

（五）进一步改善投资环境。把改善投资环境当作一项永久性工程来抓，下大力气改善投资环境，树立环境就是生产力，形成“人人都是投资环境，处处都是开放形象”的社会氛围。市委、市人民政府决定成立市级政务服务中心，作为统一的政务服务和招商引资窗口平台，凡具有行政许可职能和收费项目的部门均进入实行集中统一的一站式服务。各县区也要加快政务服务中心的组建工作。深入开展“建设诚信安顺、优化发展环境”活动，继续开展“群众评议机关”活动，抓好以政府信用为核心的信用体系建设，逐步建立和完善政府信用评价制度。加强投资环境的执法监察和各类投诉案件的查处，严格执行行政责任过错追究制度，对损害投资软环境的典型案例一查到底，争取短时间内使全市投资环境有一个大的改善，用优良环境吸引投资者。

二、围绕解决好“三农”问题，促进粮食增产、农业增效和农民增收

（一）决不放松粮食生产，稳步提高粮食综合生产能力。认真落实中央和省扶持农业发展的各项政策措施。认真贯彻执行免除农业税的政策，继续落实好对种粮农民的直接补贴、良种补贴和购置农机具补贴政策，进一步加大对农业的投入，积极争取金融机构加大对农村经济的支持力度。加强对化肥等农资价格的监管，完善涉农价格和收费公示制度，依法保障农民的合法权益。坚持走内涵式发展粮食生产的路子。着力改善品种结构，增强土壤肥力，优化区域布局，提高良种良法普及率，提高单产、增加总产。保证耕地占补平衡，加强基本农田建设，完成以坡改梯工程为主的基本农田建设任务4万亩。确保杂交水稻播种面积78万亩，杂交玉米播种面积60万亩，脱毒马铃薯种植面积10万亩。确保全年粮食播种面积234万亩，粮食产量75万吨以上。

（二）以发展生态畜牧业为重点，大力推进农业产业结构调整。一是加快建设生态畜牧业强市步伐。认真实施建设生态畜牧业强市规划，狠抓良繁体系、饲草饲料体系、动物防疫体系、畜牧执法体系建设，提高畜禽优质率、出栏率、商品率，力争今年畜牧渔业产值有新的突破。二是加快特色农产品产业化建设。以市场为导向，以大企业为带动，突出抓好农业生产基地建设，扩展“一乡一特、一村一品”，集中连片发展优质米、蔬菜、茶叶、烤烟、中药材等基地化、规模化、品牌化生产，提高科技含量和附加值，建设一批产业示范基地。确保茶叶种植面积达到10万亩以上。确保完成20万担以上的烤烟收购任务，高标准建设好红河卷烟厂烤烟基地，在此基础上力争用2-3年的时间建成30万担以上烤烟生产基地。加大对山药、山苍籽、花椒、辣椒、生姜、韭黄等特色农产品的扶持，加快建立特色农产品生产基地。三是进一步加强农业科技推广体系建设。健全乡镇农技推广网络，搞好农业适用技术培训，大力推广农业适用技术，努力提高技术入户率。充分发挥职能部门和专业技术人员在产业指导、基地培育、适用技术推广等方面的作用。努力保证公益性技术推广工作的经费需求，积极稳妥地将一般性技术推广和经营性服务分离出去，按照市场化方式运作。四是积极推进农业产业化经营。加大对龙头企业的扶持力度，引导龙头企业和农户形成生产、加工、销售的利益共同体。大力发展订单农业。加强以产地批发市场为重点的农产品流通基础设施建设，提高农产品商品率。充分发挥供销社在农业产业化经营中的作用，积极发展农民专业合作经济组织和农村专业技术协会，提高农民进入市场的组织化程度。

（三）切实加强对农村劳动力的培训，努力促进农村剩余劳动力有序转移。实行培训经费与培训转移情况挂钩，大力推广订单培训等培训方式，逐步形成政府主导、统一协调、各方参与的农村劳动力转移培训体系。实行定向输出、定点输出，引导农民自发输出，建立劳务输出基地。通过推进农业结构调整、加快乡镇企业发展和小城镇建设等途径，促进农村劳动力就近就地转移。高度重视维护农民工合法权益，取消对农民进城就业的限制性规定。鼓励工会、共青团、妇联及其他社会组织帮助农村劳动力有序转移。

（四）按照“三个基本”的要求，切实加快新阶段扶贫开发。继续落实好6个一类重点扶贫乡镇各项扶持政策，进一步做好规划，实施整村推进。切实抓好贫困地区劳动力培训和产业化龙头企业扶贫等工作，促进贫困地区加快产业结构调整。认真做好国家扶贫开发重点县三类重点扶贫乡镇的定期救助工作。进一步完善信贷扶贫工作，争取更多的扶贫信贷资金。农村基础设施建设、“两基”攻坚和农村公共卫生项目及资金要进一步向贫困地区倾斜。实行扶贫项目公示和项目决算公告制度，完善项目验收制度，加强扶贫项目和资金的审计监督，提高资金使用效益。继续坚持和完善分类指导、党政机关和领导干部扶贫联系点制度。继续抓好以青岛对口帮扶为重点的东西扶贫协作。积极探索扶贫开发工作新办法新机制，努力调动全社会力量参与扶贫帮困工作，增强贫困地区自我发展能力，切实抑制返贫。同时，支持关岭、紫云申报国家农业综合开发县。

三、加快工业结构调整，努力促进工业经济增长方式转变

（一）进一步做大做强优势产业。一是大力发展煤炭产业。按照“大煤保大电”和“煤电联营”的思路，加快电煤基地建设。上半年完成对各县区煤炭资源的进一步普查、详查、精查和规划布局工作，10月份完成开采相关规划。围绕今年开工建设100万吨以上大矿目标，继续抓好新规模矿井的建设，提高煤炭产量。进一步完善煤炭调运系统，尽快建成全市煤炭安全生产监控系统，从源头上加强煤矿的平衡生产和煤矿安全生产。加快煤化工产业发展。二是做大做强制药业。抓好中药材开发和加工，重点发展一批在国内外市场有良好前景的地道的中药制剂及民族药，认真做好药材资源的保护工作。大力扶持百灵制药、寿仙药业、顺健制药、玖久中药等企业做大做强，支持安顺中药有限公司、西秀制药厂在上半年完成GMP改造。积极引导企业建设集中联片、规范的中药材GAP种植基地。三是稳健发展高载能产业。合理引导铁合金、电解铝、水泥等产业向环保型、规模化发展，提高产业集中度和技术装备水平，延长产业链，形成产业群。四是加大技改工作力度。以完善安全生产设施、发展循环经济、提高技术水平为重点，运用先进适用技术改造提升能源、制药、化工等优势产业，提高产业素质和核心竞争力。积极争取国家和省的技改专项支持，适当增加财政技改贴息资金投入，鼓励吸引社会资金和各类投资者参与企业技改。五是大力支持军工企业发展。积极支持军工企业改革改制工作，帮助实施军民品分离政策的落实。全力帮助军工企业解决存在的困难和问题，为军工企业创造良好的发展环境。支持军工企业加快以航空、汽车、机械、精密仪器等为代表的高新技术产业快速发展，加快确定我市轿车工业生产基地，实现国防工业与地方经济共同发展。

（二）加快培育和发展特色产业。坚持培育与引进相结合的原则，进一步培育壮大牛来香、康润（华泰）、百花串等特色品牌，力争引进一批优强企业和名优品牌，促进农产品加工上规模、上档次。财政支农资金和农业综合开发资金等，要重点支持农产品加工企业基地建设、科研开发、技术服务、质量标准和信息网络建设。围绕旅游业发展，重振安顺传统轻工业。大力发展旅游商品生产，着力开发蜡染、布依地毯、地戏脸谱、波波糖、航空模型、古生物化石工艺品等产品，努力扩大生产规模，拓展市场。

（三）大力发展循环经济。发展循环经济是推进我市优先发展重点旅游区建设，走新型工业化道路和环境立市的必然选择。大力推广节能新技术、新工艺和先进经验，加强对电力、煤炭、化工、建材、冶金等行业的能源、原材料和水等资源消耗的管理，加快建立资源节约型社会。大力支持“三废”综合利用和再生资源回收利用，加快城市生活污水治理设施建设和垃圾资源化利用。认真落实《中华人民共和国清洁生产促进法》，推行重点排污企业公告制度和强制性清洁生产审核制度。

（四）依法加大资源环境保护力度。加强矿产资源的保护和管理，切实增强对矿产资源的统筹能力。严厉打击非法勘查开采，依法查处非法转让矿权、圈而不探、以探代采和破坏性开采行为，积极推行生态型开采和边采边恢复模式。全面推进探矿权、采矿权招标拍卖挂牌出让，培育和规范矿权市场。做好土地利用总体规划修编，实行最严格的耕地保护制度，严格控制建设用地增量，努力盘活土地存量，用好关闭破产企业的闲置土地，保障全市经济社会发展建设用地需要。抓紧编制地质灾害防治规划，加强地质灾害防治工作。加强酸控区、饮用水源区、重点流域以及中心城区的综合整治，向社会公布饮用水源地水质达标情况。加大工业污染防治力度，对未达标的污染企业限期实现排污达标。积极调整城乡能源结构，提高城市燃气和农村沼气普及率。

四、推进优先发展重点旅游区建设，全面提升安顺旅游竞争力

（一）大力发展旅游业，努力实现旅游业的率先突破。旅游业在我市推进历史性跨越进程中是极具基础、极具活力、最有可能率先实现突破的产业。一是深化对安顺旅游资源的认识，进一步搞好旅游发展规划，包括旅游产业发展规划、旅游景区发展规划、旅游服务人员规划等，增强深度和可操作性。二是抓住安顺作为省优先发展重点旅游区的机遇，以黄果树为龙头，紧紧抓住屯堡文化、蜘蛛人徒手攀岩等绝无仅有的资源，整体作为精品包装，打造安顺旅游的强势品牌，实现今年游客数超过400万人次、力争500万人次，旅游总收入突破10亿元。黄果树风景区要在去年游客突破百万人次的基础上，实现新的突破，带动安顺旅游业快速发展。黄果树瀑布节今年作为全省性的旅游节由国家旅游局和省政府主办，我们要做好承办工作，使黄果树瀑布节名气更大、影响更大，真正成为全省乃至全国的品牌节庆活动。三是全力做好旅游宣传和市场营销工作。按照“统一宣传口径、统一促销行动”的原则，把旅游宣传作为大文章来作，强化市级统一的宣传促销。通过在一批大城市举办安顺旅游文化周等多种宣传方式，加大宣传力度，巩固和扩大主打客源市场。以开拓韩国旅游市场为突破口，积极开拓和培育新的国内外客源市场。高度重视导游队伍的建设和培训。做好旅游精品线路推介，增加游客在安顺停留时间。做好《安顺市民文明手册》发放工作，进一步在全社会形成人人关心旅游、支持旅游发展的良好氛围。四是加大旅游基础设施建设力度。加快黄果树“六大”景点开发步伐，推进黄果树新城建设，加快生态建设步伐，把黄果树建设成为人与自然和谐统一、功能完善、设施齐全的世界级景区。努力推动黄果树形成国际会议中心。搞好龙宫景区的深度开发，完善花江大峡谷、格凸河、夜郎湖和屯堡文化旅游区的配套设施建设，完成关岭化石群国家地质公园基础设施建设，加快普里山风景区开发步伐，提升兴伟古生物化石博物馆品牌，建成开放双阳航空博览馆。积极发展自然风光游、历史文化游、休闲度假游、科考探险游、乡村旅游和工业旅游，积极发展以王若飞故居为重点的红色旅游。继续做好格凸河申报国家级风景名胜区和申报世界自然遗产工作。大力支持黄果树机场的建设和发展，依托旅游业发展民航事业。五是大力提高管理和服务水平。依法管好资源，灵活放开经营，严格按规划保护和开发旅游资源。进一步规范特许经营行为，凡是风景名胜区内实行特许经营的项目，必须经市委、市人民政府批准。积极支持各类旅行社规范、健康、快速发展，发挥其在旅游业发展中的市场主体作用。加强旅游业管理和服务的标准化、规范化建设，努力形成具有一流水平的管理服务体系。在全市开展文明景区、文明酒店、文明导游、文明公司、文明旅行社评选工作。六是充分挖掘少数民族民间文化、地方特色文化和民俗风情，与旅游发展相结合，丰富旅游内涵。

（二）围绕建设中国优秀旅游城市，进一步完善城市功能。抓住我市作为省规划的以贵阳为龙头的城市经济圈的机遇，以创建中国优秀旅游城市为目标，着力打造旅游文化城市，全面推进新一轮城市建设。进一步完善城市功能，推进以城市为中心组织区域经济发展的步伐。要严格规划，切实按照旅游文化城市来建设和培育环境，重塑历史文化名城。进一步改革城市建设投资体制，完成塔山公园、文庙环境整治、龙泉路改造等建设工程，继续抓好中华东西路、贯城河二期治理、市垃圾无害化处理、西秀新区供水管网和双阳新城区基础设施建设工程，建设王若飞生平事迹陈列馆及停车场，启动黄果树大街、虹山水库环湖路等工程项目建设，力争开工建设市污水处理工程。重视公共设施的建设和管理，重视市区和县城小街小巷的治理改造。以居民住宅为重点，加大经济适用住房建设力度，满足中低收入家庭需要。进一步搞活住房二级市场。继续做好设立市辖双阳区的申报工作，进一步加快西秀新区建设步伐，壮大中心城区规模。抓好高星级酒店和度假酒店建设，促成一批四星级以上酒店在我市落户。对现有酒店符合条件的实施星级改造和管理。

（三）以服务业为重点，全面提高第三产业发展水平。充分利用我市的立体交通运输网络，按照《安顺城市商业网点规划》，加快发展一批专业市场和旅游产品市场，扩大辐射范围。加快发展交通运输业特别是公路运输业，尽快形成市区、景区旅游交通客运网络。推动公共交通、供水排水、园林绿化、环境卫生等传统公益性服务业在竞争中发展提高。鼓励创办各种社区服务，积极发展面向生活消费的金融、保险服务，大力发展连锁经营、物流配送、电子商务等现代流通业，规范发展会计、法律、拍卖、咨询等中介服务。

五、深化把多种经济成分共生繁荣改革试验区扩大到全市的改革，增强全市经济发展活力

（一）深化多种经济成分共生繁荣试验区改革，大力发展非公有制经济。进一步解放思想，深化把多种经济成分共生繁荣改革试验区扩大到全市的认识，围绕解决影响发展全局的深层次矛盾和问题，推进体制、机制和政策的创新，创造良好的发展环境，增强全市经济发展活力。认真贯彻落实国务院《关于鼓励支持和引导个体私营等非公有制经济发展的若干意见》，实行鼓励、支持和引导非公有制经济发展的政策措施，为非公有制经济创造平等竞争、一视同仁的法治环境、政策环境和市场环境。进一步放宽社会资本的投资领域，落实非公有制经济投资的自主权，对于非公有制企业不使用政府投资建设的项目，改审批制为核准制或备案制。进一步拓宽非公有制经济融资渠道，依法协调配套使用现有支持中小企业发展的各项资金，鼓励非公有制企业通过中小企业板市场、项目融资等渠道和方式筹措资金，健全中小企业信用担保体系，逐步提高担保能力。加强非公有制经济服务体系建设，在企业创办、产权交易、技术创新、人员培训、对外合作以及法律、信息等方面为非公有制企业提供完善的服务。鼓励和支持非公有资本进入基础设施、垄断行业、公用事业以及法律法规未禁止的其他行业和领域；鼓励和支持非公有资本采取购买、兼并、控股等形式参与国有企业改制重组。支持非公有制经济更多地向农产品基地建设、农产品精深加工、生态畜牧业等方面发展，带动我市农业产业化水平的提高。引导和鼓励非公有制经济依法、规范发展教育、科技、文化、卫生、体育等社会事业。切实保护私有财产权和非公有制企业及其职工的合法权益，严肃查处各种乱收费、乱罚款和乱摊派等损害非公有制经济发展环境的行为。

（二）采取有力措施，确保实现国有企业“两个百分之百”的改革目标。进一步加大推进国有企业改革工作的力度，充分用好用足国家和省、市的有关政策，积极筹措改革改制费用，维护好职工利益，通过多种方式特别是市场化方式，确保全面实现国有企业百分之百改制、职工身份百分之百转换。加强对全市国有企业改革工作的领导、指导和协调，规范企业改革改制工作，及时研究解决改革中出现的问题，保证改革顺利推进。严格按照市关于推进国有企业改革工作的进度要求，制定考核目标，层层分解落实，对工作进展情况实行定期督查考核制度，确保目标责任落实到位。同时，积极帮助已改制重组的企业理顺关系，搞好生产经营，加快形成一批新的经济增长点。继续配合做好一批省属企业的破产关闭和破产重组工作。

（三）全面落实农村各项改革政策，推进财税金融改革。继续深化农村税费改革，进一步完善农村税费改革转移支付资金管理办法，配套推进乡镇机构、农村义务教育管理体制和县乡财政管理体制三项改革。开展乡村债务清理工作。继续深化粮食流通体制改革，巩固粮食流通企业改革取得的成果。进一步完善部门预算和国库集中收付制度。优化财政资金支出结构，确保重点支出需要。完善和规范政府采购制度，深化“收支两条线”改革。强化税收征管，加强对重点税源的监控和管理，认真搞好后续财源的培育工作。强化政府债务管理，防范财政风险。支持农村信用社改革，加快组建农村合作银行。加大安顺城市信用社改革发展力度，加强和完善内控机制，提高资产质量，加快组建地方商业银行步伐。

（四）加快发展县域经济，实现地区之间协调发展。以科学发展观为指导，按照构建和谐社会和“五个统筹”的要求，结合国家产业政策，科学制定今后五年我市的发展目标，完成“十一五”规划及相关专项规划编制工作。加强和改进对县区工作的领导，科学设定市与县区、县区与乡镇（办事处）的经济管理体制，进一步规范市与县区、县区与乡镇（办事处）的经济管理职责权限，提高各级履行职责、领导发展的能力，充分调动和发挥市、县、乡三级独立作战和加快发展的积极性和主动性。加快发展县域经济，加快以县城为重点的小城镇建设，推进城镇化步伐。健全和完善乡镇（办事处）财政管理体制，建立激励与约束相结合的发展机制。指导乡镇（办事处）结合资源特点，科学定位发展方向，形成自己的特色产业。继续开展并完成第一次全市经济普查工作，组织实施好人口抽样调查工作。

（五）坚持“引进来”和“走出去”相结合，努力提高开放水平。抓住我国与东盟建立自由贸易区的机遇，发挥我市作为南贵昆经济区主要中心城市的有利条件，积极参与“泛珠三角”等区域经济合作，加强与南贵昆经济区其他城市和东部沿海省区的合作，着力向外宣传推介我市的优势产业、优质资源、优秀品牌，充分利用两个市场、两种资源吸引更多的外来投资。支持有条件的企业发展多种形式的对外经贸关系。积极争取并用好国家中小企业国际市场开拓资金和国家西部地区外经贸发展促进资金，切实用好省外经贸发展资金和出口信用担保基金，加大对我市相关企业产品出口的支持力度。加强与周边市州地在交通建设、能源开发、水利建设等方面的合作。认真做好各项服务工作，确保中国科学院技术科学论坛和地方科技咨询活动在我市成功举办。

六、全面发展各项社会事业，努力构建社会主义和谐社会

（一）大力实施人才战略，抓好各类人才队伍建设。牢固树立人才资源是第一资源的观念，加强多层次人才队伍建设，切实抓好行政管理人才、企业经营管理人才、专业技术人才、农村适用人才、少数民族人才和妇女人才的培养、选拔和使用，努力在全社会形成人尽其才、人才脱颖而出的良好局面。健全科学的人才评价机制，把各类人才的积极性和创造性引导好、保护好、发挥好。积极配合省实施“四个一工程”和青年科技人才、学科带头人培养工作。继续开展培养“一村一名大学生”工作。加大高层次人才引进工作力度。整合人才资源，优化人才配置，促进人才在产业、地区、城乡间合理分布。不断完善对人才的激励机制，认真落实关于人才的各项政策待遇，努力做到用事业留人，用感情留人，用适当的待遇留人。全面推行事业单位人事制度改革，进一步调动和发挥广大专业技术人员的积极性和创造性。做好全国全省劳动模范、先进工作者的评选工作，进一步营造劳动光荣的氛围。

（二）大力实施科教兴安战略，加快发展科技教育事业。坚持把基础教育作为教育工作的重中之重，提高基础教育的整体质量和水平，确保紫云“两基”达到预期目标，认真做好已通过“两基”验收县区的巩固提高工作。大力发展普通高中、民族教育、高等教育、成人教育和农村中小学现代远程教育，鼓励和引导民办教育健康发展。高度重视职业教育，支持安顺职业技术学院及一批职业培训学校加快发展。继续做好“安顺学院”申报工作。推进市二中新校区建设。确保农村税费改革转移支付资金用于农村教育的比例不低于50%。扩大农村义务教育阶段贫困生“两免一补”政策的实施范围，保证学生不因贫困而辍学。关注城市家庭经济困难学生、务工农民子女的义务教育。继续实施好“西部开发助学工程”。继续治理学校乱收费，严格实行义务教育阶段“一费制”和高中招收择校生“三限”政策。加强和改进未成年人思想道德建设和大学生思想政治教育，努力构建学校教育、社区教育、家庭教育相互结合的德育教育体系。大力推进科技创新。完成中长期科学和技术发展规划的制定工作。集中力量抓好省市重大科技项目的实施，推广一批见效快、覆盖面广的适用技术，有重点地发展高新技术。加强农业科技创新，进一步加大对农业科研和农业科技成果转化的支持力度。抓好以企业为主体的技术创新体系建设，支持企业建立和完善技术研发创新平台。在中药现代化、农业关键技术攻关、石漠化治理等重点领域，加快科技集成创新、成果转化和产业化步伐。抓好科学技术普及工作，促进经济增长方式转移到依靠科技进步和提高劳动者素质的轨道上来，提高科技进步对经济增长的贡献率。切实加强知识产权保护，提高企业运用知识产权的能力和水平。

（三）抓好公共卫生服务体系建设，加强人口和计划生育工作。继续加快公共卫生体系建设，进一步完善突发公共卫生事件应急处理系统，初步建成疾病预防控制体系，完善医疗救治体系、卫生监督体系建设。深化城镇医疗卫生服务体制改革，提高重点综合性医院的发展水平，大力发展社区卫生服务，积极探索向乡镇和社区派出卫生监督员的新路子。改革和完善农村卫生投入机制，加大对村卫生室建设的支持力度。加大城市对农村卫生工作的支持，建立农村贫困家庭医疗救助制度，采取有效措施解决农村缺医少药和因病致贫、因病返贫问题。加强计划免疫工作，全面落实重大传染病和地方病防治措施。继续抓好平坝县新型农村合作医疗试点工作。全面推行药品集中招标采购制度，规范医疗服务价格，严肃查处医疗服务中的违法违纪行为。大力开展和实施“食品药品放心”工程，努力营造消费者满意的安全环境。巩固计划生育“村为主”，全面推行村（居）民自治。完善和落实对农村计生家庭的奖励扶助政策，推进实施“少生快富”工程。抓好西秀区、普定县巩固“全省优质服务先进县区”工作。搞好人口发展战略研究，实施人口出生缺陷干预，高度重视治理出生人口性别比升高问题。继续加强流动人口的管理和服务。确保符合政策生育率达到90%以上。

（四）推进文化广播电视等事业全面发展，不断满足人民群众的需要。重视市级公益文化设施建设，加快文化广场建设步伐。继续推进以县级图书馆、文化馆、档案馆和乡镇文化站为重点的基层文化设施建设，力争用五年左右的时间逐步形成覆盖全市的比较完备的公共文化服务体系。重视保护和扶持重要文化遗产和优秀民间文化。切实做好村村通广播电视工程和电信村村通工程。积极推进广播电视网络建设和升级改造，认真落实广播电视导向安全和安全播出的各项措施。积极推进全民健身运动，做好市民健身中心建设的启动工作。提高竞技体育水平，做好2006年全省青少年运动会的选拔训练工作。全面贯彻党的民族宗教工作基本方针和政策，认真执行《宗教事务条例》等民族宗教法律法规，加快民族地区发展，保持宗教稳定。积极支持国防建设，努力实现国防建设与经济建设协调发展。加强国防教育，抓好民兵预备役队伍建设。认真实施《妇女儿童发展纲要》。进一步做好老干、档案、史志和外事、侨务、对台工作，支持工会、共青团、妇联、科协、残联、红十字会等人民团体依法按章开展工作。

（五）继续加强就业再就业工作，不断健全社会保障体系。把调整经济结构、转变经济增长方式与促进就业再就业统筹谋划，注重发展就业容量大的行业，逐步形成有利于扩大就业的经济结构和增长模式，不断提高经济增长对就业的吸纳能力，在发展中解决就业问题。认真落实促进就业再就业的各项政策措施。加快《再就业优惠证》的审核发放，进一步发挥小额贷款在促进再就业工作中的作用。对吸纳下岗失业人员和开发公益性岗位促进“4050”人员再就业的企业落实有关优惠政策。加强政府就业指导和服务，抓好就业培训，推动就业服务制度化、社会化和专业化。发挥街道、社区劳动保障工作站的优势，建立健全失业人员登记制度，加强对城镇失业率的监测。不断完善市人才市场、劳动力市场的功能，发挥其在解决大中专毕业生、复转军人、新增劳动力等人员就业方面的作用。继续巩固“两个确保”100%的成果，加强“三条保障线”之间的衔接。做好改制企业职工的养老保险接续工作，做实企业职工基本养老保险个人账户，依法把应保未保的职工纳入社会保险。以非公有制企业、个体户、灵活就业人员为重点，扩大社会保险覆盖范围。加大社会保险费的征缴力度，提高征缴率。努力提高工伤保险参保率。抓好城市“低保”的规范化管理，建立市、县、街道三级“低保”业务处理网络系统，把所有符合条件的城镇困难群众纳入“低保”范围，做到应保尽保。稳步推进农村救济救助工作，积极探索建立农村社会保障体系。加快推进企业退休人员的管理服务社会化，逐步将其纳入街道、社区进行管理和服务。

（六）大力加强社会主义精神文明建设，努力提高全市文明水平。深入贯彻落实《公民道德建设实施纲要》，深入开展爱国主义、集体主义、社会主义思想教育，加强社会公德、职业道德和家庭美德建设，在全社会倡导爱国守法、明礼诚信、团结友善、勤俭自强、敬业奉献的公民道德规范，大力培育弘扬民族精神和时代精神。深入开展学习型城市、学习型机关创建活动，深入开展文明城市、文明村镇、文明社区、文明行业、文明单位、文明机关、“四在农家”等群众性精神文明创建活动，大力提倡移风易俗，积极引导城乡居民形成科学健康文明的生活方式，努力提高全社会的文明水平。深入推进“卫生与秩序”创建活动，在巩固和提高市区、县城创建工作成效的基础上，把创建活动延伸到乡镇，建立科学、规范、操作性强的考评体系，将创建工作纳入对各县区的目标考核。继续搞好“三下乡”活动和“四进”社区活动。认真组织实施好“百县千乡宣传文化工程”，抓好县级宣传文化中心、乡镇精神文明活动中心和村文化活动室建设工作。广泛开展“双拥”共建活动，争创“双拥模范市”。

（七）加大社会治安综合治理力度，切实维护社会稳定。全面落实维护社会稳定工作责任制，强化社会治安综合治理，扎实开展“平安安顺”创建活动，严密防范和打击“法轮功”等邪教组织，依法严厉打击各类刑事、经济犯罪。认真贯彻落实《反分裂国家法》。深入推进“四五”普法工作，增强公民法律意识 。认真落实《信访条例》，做好集中处理信访突出问题和群体性事件工作，加强和完善信访联席会议制度，依法维护信访秩序，及时化解各种社会矛盾。认真解决征地、移民、拆迁、企业改制等方面存在的损害群众利益、影响社会稳定的问题。加大清理拖欠民工工资力度，确保不发生新的拖欠，对存在拖欠民工工资的，严格按照有关规定督促限期兑付。进一步加强清理整顿农村合作基金会等“两会一部”的工作力度，采取多种方式解决好对储户遗留的资金兑付工作。继续做好困难企业军转干部的解困工作。

加强政府行政能力建设

努力提高行政能力，是各级政府贯彻党的十六大和十六届三中、四中全会精神，加强党的执政能力建设的具体体现，是一项长期而重要的任务。政府系统各级党组织及广大党员，要认真开展保持共产党员先进性教育活动，认真贯彻落实胡锦涛总书记视察贵州时的重要讲话精神，自觉做到“三个新”，落实“四个要求”，认真解决政府系统党组织和党员队伍中存在的突出问题，带头确保在“提高党员素质，加强基层组织，服务人民群众，促进各项工作”上取得实效，不断增强创造力、凝聚力、战斗力，切实提高政府行政能力。

一、坚持依法行政，切实转变政府职能

牢固树立依法行政的观念，严格遵守宪法和法律，深入贯彻实施《行政许可法》，继续深化行政审批制度改革。按照合法行政、合理行政、程序正当、高效便民、诚实守信、权责一致的要求行使权力。积极推进综合执法，实施相对集中行政处罚权和相对集中行政许可权制度，切实解决多头执法、交叉执法、执法不公的现象，不断提高依法行政的能力和水平。进一步增强按市场经济规律管理经济的能力，提高运用经济和法律手段调节经济运行的意识和能力。在继续加强经济调节和市场监管的同时，更加注重社会管理和公共服务职能，把财力物力等公共资源更多地放到社会发展和解决人民群众切身利益的问题上来。进一步解决好政府“越位”、“缺位”和“错位”的问题，推进政企分开、政资分开、政事分开，把政府不该管的事交给企业、市场和社会组织，为市场主体创造良好的发展环境。

二、坚持执政为民，全心全意为人民服务

牢固树立正确的政绩观。讲实话、察实情、办实事、求实效，坚持深入基层、深入群众，把维护好、实现好、发展好广大人民群众的根本利益作为政府工作的出发点和落脚点。创新政府管理方式，加强协调，密切配合，提高行政效率，更好地为基层、企业和社会公众服务。推进电子政务建设，降低行政成本，促进政府办公网络化、信息化，提高政府公信力。进一步推进政务公开、村务公开和厂务公开，加大政务信息发布力度，增强工作透明度。进一步健全社会公示、社会听证和专家咨询等制度，让人民群众更广泛地参与公共事务管理，拓宽群众对行政事务知情、参与和监督的渠道。切实提高处置突发事件和保障公共安全的能力。认真制定《安顺市人民政府突发公共事件总体应急预案》以及相关子预案。全面落实安全生产责任制和安全事故责任追究制度，加大安全生产执法力度。

三、坚持民主执政，不断提高科学决策水平

各级政府要自觉接受同级人大及其常委会的法律监督和工作监督；自觉接受同级政协的民主监督；认真听取民主党派、工商联、无党派人士和各人民团体的意见和建议；自觉接受新闻舆论和社会公众的监督；重视人民群众对政府机关及其工作人员的监督。支持人民法院和人民检察院依法独立行使职权。支持监察、审计部门依法独立履行监督职责。进一步改革和完善决策机制，坚持民主集中制原则，坚持重大问题集体决策制度，坚持集体领导下的分工负责制，推进决策的科学化和民主化。进一步加强责任制度、督查制度、考核制度和报告制度建设，对行政过错依法追究责任，保证各项决策执行不走样。今年，市政府对事关全市发展的重大工作实行调度会议制度，用制度来保证各项工作落到实处。

四、坚持廉洁从政，切实加强政风建设

认真抓好党风廉政建设和反腐败工作，严格遵守“四大纪律”、“八项要求”，重点抓好政府系统廉政工作八个方面的改革和制度建设。严厉惩处腐败分子，严肃查处顶风违纪行为，坚决纠正损害群众利益的不正之风。严禁形式主义、官僚主义、弄虚作假，严禁搞脱离实际、劳民伤财的“政绩工程”和“形象工程”。严肃财经纪律，进一步加强和完善对政府投资项目建设、交通建设、扶贫资金和工程招投标等重点领域和关键环节的管理，所有政府性投资建设项目一律实行“代建制”，所有经营性土地一律实行招标拍卖挂牌出让。继续精简会议和文件，切实改进会风和文风。严格规范和控制各种检查、评比、达标活动。高度重视“群众评议机关”活动中提出的意见和建议，结合自身存在的问题认真加以整改，推动政风建设。加强对公务员的教育、管理和监督，加大公务员培训力度，努力建设一支人民满意的公务员队伍。

各位代表，做好今年的各项工作，任务艰巨，意义重大。让我们在以胡锦涛同志为总书记的党中央领导下，以邓小平理论和“三个代表”重要思想为指导，按照省委、省人民政府的总体部署，在市委的统一领导下，在市人大、市政协的监督和支持下，凝聚各族各界群众的智慧和力量，抓住机遇、用好机遇，同心同德、加快发展，努力完成今年经济社会发展各项目标任务，为加快推进安顺小康建设和富民兴安步伐，实现经济社会历史性跨越而努力奋斗！
